# Fred Goldberg machines in the pathogenesis of *Mycobacterium tuberculosis*

**DOI:** 10.1016/j.jbc.2026.111373

**Published:** 2026-03-16

**Authors:** K. Heran Darwin

**Affiliations:** Department of Microbiology, NYU Grossman School of Medicine, New York, New York, USA

**Keywords:** *Mycobacterium tuberculosis*, proteasome, Clp protease

## Abstract

Regulated proteolysis is found in all forms of life, from bacteria to humans, and plays essential roles in all areas of physiology. The bacterial pathogen *Mycobacterium tuberculosis* has three, chambered ATP-dependent proteases needed to cause lethal infections in animals, making them attractive targets for drug development. Here, I review the latest developments in what is known about how caseinolytic proteases and proteasomes function to contribute to the virulence of one of the world's deadliest pathogens.

I was once asked, “Why does *Mycobacterium tuberculosis* (*Mtb*) need a Rube Goldberg machine to degrade a protein?” My first response was, “We don't ask why questions in biology,” followed by “What is a Rube Goldberg machine?” Rube Goldberg became famous for his fantastical, overly complicated devices with many moving parts, all to accomplish a single, simple task like flipping a light switch or turning on a toaster. While I did not know the name Rube Goldberg, I was aware of his eponymous machines, and that they are a euphemism for something that is way more complicated than it needs to be. The person who asked me the “why” question perhaps had a point when it comes to protein degradation.

Another Goldberg was a pioneer in the field of regulated proteolysis and grew interested in the prospect of not only studying proteases but also in finding new treatments to cure old diseases. In addition to mammalian protease pathways, Alfred Goldberg, who went by “Fred,” appreciated that prokaryotic and eukaryotic degradation systems share sequence and biochemical similarities. Among the microbes he started to focus on was *Mtb*, a bacterial species that causes the pulmonary disease tuberculosis (TB).

A staggering statistic about *Mtb* is that it is estimated to have infected about one-third of the world's population. While this number is debated (1), the number of people who die of TB—about 1 to 2 million per year—is undeniable (2). TB is often thought of as a disease of the past; however, recent outbreaks in the United States are a reminder that we lack a durable vaccine. In addition, antibiotic-resistant *Mtb* strains are an increasing problem globally. Given the essential roles of regulated proteolysis, there has been high interest in investigating the roles of proteases in this devastating pathogen in order to find new ways to eradicate TB.

## ClpP, the original gangster

Caseinolytic protease (Clp or ClpP), once also known as "Ti," is one of the best-studied bacterial proteases, or "original gangster," which was codiscovered by the Maurizi, Gottesman, and Goldberg groups (3, 4). The name ClpP arose from its activity on casein used in the early days of protease discovery. ClpP is conserved in almost all known bacterial species (reviewed in (5)) and is also found in mitochondria (6) and chloroplasts (7). Fourteen ClpP subunits form two stacking rings resembling a barrel with polypeptide access points on either side that are normally closed. Each ClpP subunit has a catalytic serine; therefore, a canonical ClpP complex has potentially 14 active sites. To degrade proteins, a hexameric ATPase chaperone must engage the ClpP barrel to open and drive substrates into the proteolytic chamber (reviewed in (8)). In *Escherichia coli* and other species, substrates can either have intrinsic sequences (9), polyalanine tails added by RqcH (10) or arginine phosphorylation (11, 12) which recruit them to ATPase chaperones like ClpX, sometimes with the help of adaptors like ClpS (13). Most remarkably, a small peptide called SsrA, encoded by a transfer–messenger RNA, can be added to stalled polypeptides in ribosomes, aiding in their release from the ribosome and targeting them to ClpP for destruction (14).

The prevalence of Clp proteases and their accessory factors across domains of life have made them an active area of research for decades as well as an appealing drug target. About a decade ago, potent antibiotics called acyldepsipeptides (ADEPs) were identified to directly bind to ClpP subunits and stimulate substrate degradation in the absence of ATPase chaperones (15, 16). Whereas loss of ClpP function does not always inactivate bacterial viability, constitutive ClpP activity caused by ADEPs is deadly for most microbes tested so far, making ADEPs a particularly exciting area for drug development against pathogens like *Mtb*.

**Figure 1:**
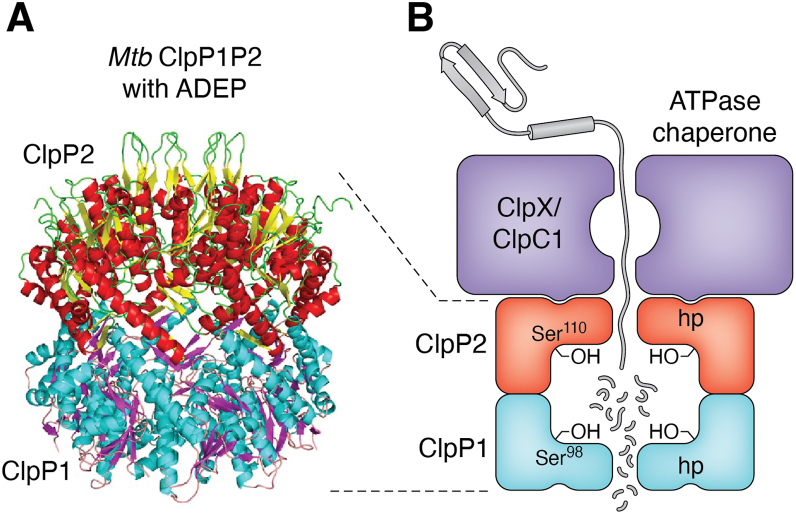
***Mtb* ClpP is a heterotetradecameric complex.***A*, crystal structure of *Mtb* ClpP1P2 with ADEP (Protein Data Bank code: 4U0G) (15). *B*, schematic of ClpP1P2 with an adaptor ATPase. Figure adapted from Ref. (21). *Mtb* ClpP1P2 forms a heterotetradecamer where ClpP2 interfaces with an ATPase chaperone. ClpP1, but not ClpP2, protease activity is essential for degradation. ADEP, acyldepsipeptide; ClpP, caseinolytic protease; *Mtb*, *Mycobacterium tuberculosis*.

Several bacterial genera, including mycobacteria, have two *clpP* genes in an operon, *clpP1 clpP2*, that encode interacting subunits (reviewed in Ref. (5)). The presence of two highly similar ClpP alleles baffled the field for several years. While recombinant mycobacterial ClpP1 and Clp2 can be individually produced in *E. coli*, their activities are low; however, when combined, the subunits can assemble into highly active, heterotetradecameric complexes (17). The Goldberg laboratory predicted that *Mtb* ClpP1 and ClpP2 would each form homoheptameric rings that interact, a model that was ultimately shown in a crystal structure (15) (Fig. 1). Moreover, the Sauer laboratory found ADEP interacted specifically with the ClpP2 ring, yet could open both sides of a ClpP1P2 tetradecamer (15). This work strongly suggested ATPase chaperones like ClpX bound the ClpP2 subunits in *Mtb*. Ultimately, the Weber-Ban group showed that hydrophobic patches on *Mtb* ClpP2 are critical for ClpX- or ClpC1-dependent substrate degradation, supporting the role of ClpP2 as a conduit between the chaperone and active protease subunits (18).

Unlike ClpP in bacterial species like *E. coli*, *Mtb* Clp protease is essential for viability *in vitro* and in mice (19), making it an attractive drug target for either inhibition or hyperactivation. In addition, both ATPase chaperones, ClpX and ClpC1, are essential for *Mtb* viability under all conditions tested so far (20, 21). The unusual biochemistry of *Mtb* ClpP raised additional questions, including, are all active sites required for activity? With the development of CRISPR interference in *Mtb* (22), the Rock laboratory was able to test several critical hypotheses about the ClpP1P2 complex. First, they showed that the active site serines in ClpP2 are dispensable for growth *in vitro* and in mice, further supporting the primary function of ClpP2 is for chaperone interactions (21). In addition, they showed that hydrophobic patches on ClpP2, but not ClpP1, are essential for chaperone interactions.

The essentiality of ClpP1P2 in mycobacteria may have been unexpected, given that ClpP is not essential in numerous other pathogens (reviewed in Ref. (23)). *Mtb* lacks the ATP-dependent Lon and HslVU (ClpQY) proteases, which might indicate ClpP1P2 is needed to do “double duty” in this species. However, *Mycolicibacterium smegmatis*, which is a nonpathogenic relative of *Mtb*, encodes Lon and still requires ClpP1P2 to grow (19). As in many other well-characterized ClpP systems, *Mtb* has SsrA protein tagging that is needed for the degradation of polypeptides stalled in ribosomes (“ribosome rescue”). Green fluorescent protein fused to SsrA is delivered to ClpP1P2 in *Mtb*, confirming SsrA marks proteins for degradation in this species (19). Importantly, SsrA is essential in *Mtb* (24), suggesting the frequency of ribosome stalling is high enough to be lethal to mycobacteria if left unresolved. Interestingly, the ClpP adaptor SmpB, which recognizes SsrA-tagged proteins, is not always essential in *Mtb* (24), suggesting there are other SsrA-binding proteins in mycobacteria.

A proteomics approach to identify the “ClpP/ClpC1-ome” was reported for *Mtb* strains in which expression of *clpP2* or *clpC1* was knocked down (25). Because of the essentiality of these genes and the duration of *clp* repression in this study (4 days), it is a near certainty that substantial transcriptional changes occurred in these bacteria, making it a challenge to discern transcriptional *versus* post-transcriptional effects on protein abundance. Notably, many of the proteins that increased in the knockdown strains are known substrates of the *Mtb* proteasome system (26). Nonetheless, the Agarwal group analyzed the sequences of 219 proteins that accumulated in both the *clpP2* and *clpC1* knockdown strains and identified the presence of “disorder-promoting residues” that are important for initiating proteasomal degradation in eukaryotes (27). They further showed that a predicted disorder-promoting residue in the heat shock protein 20 (Hsp20) (Rv0251c) is required for interacting with ClpC1 *in vitro* and for its degradation in *M. smegmatis* (25), supporting a role for unstructured termini in targeting proteins for proteolysis.

While protein disorder might be needed to initiate degradation, it is likely insufficient to target substrates to their respective ClpP accessory factors. There is no conspicuous homolog of RcqH needed for polyalanine tail addition in *Mtb*, but *Mtb* encodes several protein kinases that could potentially assist in targeting proteins to ClpP–chaperone complexes. Numerous proteins are phosphorylated in *Mtb* (28, 29) making phosphorylation a reasonable mechanism for targeting proteins for degradation.

Recognizing the potential issues with analyzing substrates for an essential protease, the Weber-Ban group took a different approach to find ClpC1 substrates by using a heterologous, *E. coli* two-hybrid system (30, 31). In this work, 25 *Mtb* toxin–antitoxin (TA) proteins were enriched among ClpC1 interactors. A TA system typically includes a protein that is an enzyme (the “toxin”) and an antitoxin protein or RNA that inhibits the toxin's activity or expression. TA systems are often regulated by proteases, and *Mtb* is no exception (reviewed in Ref. (32)). *Mtb* is unusual for encoding over 80 TA systems in contrast to fewer than 10, so far, in *M. smegmatis*. TA systems are classified into seven groups, with type II TA systems being the best characterized, where the antitoxin is a protein that inhibits the activity of the toxin. Studies of the first identified TA systems in other bacterial species showed that the differential stability of TA proteins is due to their regulation, typically by ClpP or Lon proteases (reviewed in (33)). Antitoxins are typically the substrates of these proteases, releasing their cognate toxins. This model was supported by the Ziemski *et al*. (31) study, which showed that the antitoxins, VapB20 and RelE1, but not their associated toxins, VapC20 and RelB1, respectively, are degraded by ClpP1P2/ClpC1. Interestingly, only a single proposed antitoxin, VapB47, was found in the proteomics study (25) but not identified in the two-hybrid screen (31).

While it is unknown which, if any of the identified TA systems, is individually required for virulence, several TA systems contribute to *Mtb* pathogenesis (34, 35, 36); therefore, it would not be surprising if the essentiality of ClpP1P2 is partly because of its regulation of numerous TA systems.

## Proteasomes: Fred's chambers of doom

Fred used to talk with some mild mortification about a review he contributed to *Scientific American*, where he referred to the proteasome as a “chamber of doom” (37). This term has lived rent free in my head for years as an apropos analogy for more than one reason: proteasomes are not only needed to “doom” proteins for destruction, but they are also essential for *Mtb* to doom animals to death.

**Figure 2:**
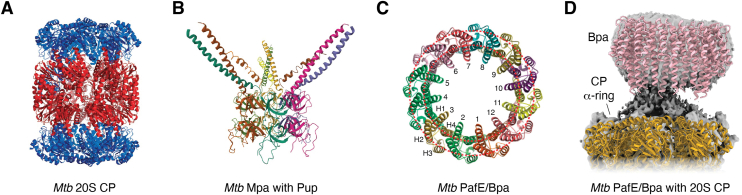
**Mycobacterial proteasome core protease and activators.***A*, crystal structure of *Mtb* PrcBA 20S CP (PDB code: 3MIO). Each ring is composed of seven subunits of either the α-subunits or PrcA in *cyan* or the β-subunits or PrcB in *red*. The α-subunits form closed gates on either end of the proteolytic chamber where the active site threonines reside. *B*, crystal structure of *Mtb* Mpa (amino acids 1–234) with the C-terminal half of Pup (PDB code: 3M9D). Six monomers form a gapped ring made of a trimer of dimers. The C-terminal half of Pup interacts with a pair of coils. Only one Pup can interact at a time with Mpa. *C*, crystal structure of *Mtb* PafE–Bpa (PDB code: 5IET). Twelve PafE–Bpa molecules form a single ring that is sufficient to deliver unfolded or specific folded proteins into a proteasome core. *D*, crystal structures of PafE–Bpa (PDB code: 5LFJ) and the 20S CP (PDB code: 5LZP) fitted to a cryo-EM map (92). CP, core particle; *Mtb*, *Mycobacterium tuberculosis*; PDB, Protein Data Bank.

Genes encoding proteins with high similarity to eukaryotic proteasomes were noticed almost 30 years ago when the first genome sequence of *Mtb* was reported (38), and crystal structures confirmed that bacterial proteasomes look nearly identical to those from Archaea and Eukarya (Fig. 2) (39, 40, 41, 42, 43). Deletion of the proteasome core protease gene, *prcBA*, from *M. smegmatis*, has no effect on bacterial viability under any condition tested (44). Several years later, a genetic screen identified mutations in *Mtb* genes annotated as “proteasome components” to be important for nitric oxide (NO) resistance and virulence (45, 46). These genes, *pafA* (proteasome accessory factor A, Rv2097c) and *mpa* (mycobacterial proteasome ATPase, Rv2115c, also known as ARC in nonmycobacteria), encode homologs of glutamine synthetase (GS) (47) and proteasomal ATPases, respectively. Mpa/ARC forms a hexameric gapped ring that facilitates degradation (46, 48, 49, 50, 51, 52), and PafA is a ligase that attaches a small protein called Pup (prokaryotic ubiquitin-like protein, Rv2111c) to proteins destined for proteasomal degradation (53, 54, 55). These components established the Pup–proteasome system (PPS).

While the proteasome core proteins and associated ATPases share substantial structural similarity with their eukaryotic counterparts, the mechanisms by which proteins are doomed for degradation differ vastly and likely emerged by convergent evolution. Ubiquitin is a 76 amino acid, highly structured small protein that is post-translationally attached to substrates to target them for degradation, and also plays countless nondegradative roles in eukaryotic physiology (reviewed in Ref. (56)). Over 1000 proteins are involved in ubiquitylation in mammals, whereas in stark contrast, *Mtb* Pup is a 64 residue, mostly disordered protein that requires only two enzymes for “pupylation” (50, 54, 57, 58, 59). In addition to PafA, a homologous protein called deamidase of Pup (Dop) converts the carboxyl (C) terminus of Pup from glutamine (Pup_Gln_) to glutamate (Pup_Glu_) (59). The Iyer group showed PafA and Dop resemble GS (47) and, like GS, PafA uses ATP to phosphorylate the terminal side-chain carboxylate of Pup_Glu_. This phosphorylated Pup intermediate is then attacked by a side-chain amino group on a substrate lysine, forming an isopeptide bond (53). For a comprehensive comparison between the Pup and ubiquitin systems, I refer readers to (56, 60).

Almost half the Pup-containing bacterial species encode Pup that ends with glutamate, indicating Dop has additional functions. Eventually, it was determined that Dop breaks Pup–substrate bonds to rescue proteins from degradation as well as recycle Pup in a process called “depupylation” (61, 62, 63, 64). Given the challenges of crystallizing a mycobacterial Dop, the Weber-Ban team solved the structure of Dop from *Corynebacterium glutamicum*, a nonpathogenic actinobacterium species (65). They showed that ATP is hydrolyzed to ADP in its active site and propose that the freed phosphate helps coordinate water to hydrolyze the amide bond in either Pup_Gln_ or between Pup and a substrate (66, 67). Using an activity-based probe where Pup has a C-terminal azido group, Burns *et al.* (62) found that a conserved aspartate in *Mtb* Dop could act as a nucleophile (68). Mutation of this proposed catalytic aspartate inactivates Dop in *Mtb* and *in vitro* (63). Based on this result, we proposed a model where an anhydride intermediate forms between Dop and Pup that is resolved with water. Unlike aspartate proteases that coordinate water to hydrolyze amide bonds, direct aspartate nucleophiles had never been described for proteases, which would make Dop highly unusual if the mechanism were correct. A structure of Dop interacting with a pupylated substrate might finally reveal how Dop catalyzes depupylation.

Given the high structural similarity between PafA and Dop—including an active site aspartate—it was possible that one enzyme could catalyze the other's reaction. Indeed, Zhang *et al*. (69) found that PafA is an efficient depupylase that requires its conserved aspartate for activity and can use ADP and phosphate instead of ATP for catalysis. Moreover, PafA can depupylate a protein and immediately transfer this Pup to a different protein (69, 70). It is unknown if this “transpupylation” occurs in bacteria or is required for *Mtb* pathogenesis, both of which are challenging hypotheses to test.

Over 60 proteins are known targets of pupylation based on proteome-wide studies (26, 71, 72) as well as from work characterizing proteins linked to proteasome-associated phenotypes. For example, work on the *Mtb* proteasome started because a genetic screen found defective proteasome activity results in increased sensitivity to NO, a free radical gas made by infected macrophages to control bacterial growth (45, 73). This hypersensitivity was caused by the accumulation of a single protein called “lonely guy” (Log, Rv1205) that had not been identified in any of the “pupylome” studies (74). Log accumulation results in the hyperproduction of hormones called cytokinins, which are adenine-based molecules that affect plant development (reviewed in Ref. (75)). While cytokinins are not toxic, their breakdown products include aldehydes that can synergize with host-derived molecules like NO and copper to kill *Mtb* (74, 76). Pup-dependent proteasomal degradation is also required for the degradation of HrcA (Rv2374c), a repressor of the essential Hsp60 regulon that includes *groES*, *groEL1*, *groEL2*, and *ruc* (Rv0991c) (77, 78, 79). Under routine culture conditions, *mpa* mutants have subtle growth defects (45) but cannot grow in minimal medium supplemented with the inorganic nitrogen source nitrate; this phenotype is due to the requirement of the Hsp60 system to ensure robust activity of the NirBD nitrite reductase complex (77).

As observed with ClpC1, several TA proteins are proposed proteasome substrates. Several TA proteins were found in a Pup pulldown experiment, although only one is a confirmed *in vivo* pupylated protein (PhoH2, Rv1095) (26, 80). The toxin VapC4 (Rv0595c), encoded in the *vapBC4* TA operon, was not identified in any pupylome study but can nonetheless be pupylated *in vitro* (81). This fascinating toxin is a nuclease that specifically targets a single cysteine tRNA to stall translation of cysteine-rich proteins, triggering an oxidative and copper stress response that increases cysteine synthesis (82). In *M. smegmatis*, cysteine-rich small proteins encoded upstream of cysteine biosynthetic genes act as cysteine sensors. It was proposed that when cysteine levels are high, an RNA secondary structure or riboswitch forms to prevent translation of cysteine synthesis genes; when cysteine is deficient, a different RNA structure forms that allows translation of downstream genes to allow replenishment of cellular cysteine (83). The Woychik group proposed that induction of VapC4 activity mimics cysteine deficiency by causing stalling at cysteine codons to promote increased expression of cysteine synthesis genes. It was further proposed that this process might mitigate oxidative or copper-induced stress that requires increased cysteine levels (82). Importantly, deletion of *vapBC4* results in reduced bacterial growth in guinea pigs, supporting a critical role for this toxin in *Mtb* pathogenesis (36). It is unknown if the antitoxin VapB4 is a substrate of proteolysis, which would be typical of canonical TA regulation, or what signals liberate active VapC4.

*mpa*, *pafA*, and *dop* mutants can persist in animal tissues but fail to kill immunocompetent mice (46, 63, 84). In contrast, strains lacking the proteasome core gene *prcBA* are rapidly cleared from mouse tissues (85, 86). Deletion of the proteasome core protease has far more deleterious effects on *Mtb* viability than disruption of Mpa or the pupylation enzymes, and this phenotype was ultimately determined to be due to the presence of a second activator. An unusual dodecameric ring with no enzyme activity, called proteasome accessory factor E (PafE) or bacterial proteasome activator (Bpa), efficiently and, remarkably, in the absence of ATP, delivers both nonspecific unfolded and specific folded proteins into proteasome cores (87, 88, 89, 90, 91, 92). Mpa/ARC and PafE–Bpa share a C-terminal, four amino acid glycine–glutamine–tyrosine–leucine motif that is essential for proteasome activation (87, 88, 93). Only two endogenous substrates have so far been confirmed: *Mtb* HspR (heat shock protein repressor; Rv0353) and ParP (partitioning with the proteasome; Rv3213c), which can be degraded by PafE–Bpa and proteasomes *in vitro* (88, 90, 92, 94). ParP is homologous to proteins involved in chromosome partitioning, but its function is unknown, whereas HspR is a well-characterized transcriptional repressor of the essential Hsp70 stress response system that includes DnaKJ and ClpB. In the absence of PafE–Bpa, HspR cannot be degraded to derepress expression of the *hsp70* regulon, leading to defective bacterial growth *in vitro* and *in vivo* (88). *In vitro* growth can be restored by suppressor mutations in *hspR*, which leads to the constitutive expression of the *hsp70* regulon (94). Fortunately, this increased *hsp* expression does not make bacteria more virulent and actually attenuates *Mtb* in mice (95).

There is promise in finding inhibitors that specifically act on bacterial proteasomes and not on mammalian proteasomes, giving hope that these enzymes could one day be targeted for TB drug development (96, 97). A challenge in TB therapeutics is finding molecules that can penetrate tissues and structures called granulomas. For example, the unusual activity of Dop, along with a robust *in vitro* activity assay, provided an opportunity to find inhibitors (98). While inhibitory molecules were found, none penetrates intact *Mtb*, re-emphasizing the uphill battle in TB drug development (99).

## It is complicated

Fred Goldberg was a biochemist through and through, but before he chose research, he was training to be a physician. Despite leaving medicine, he nonetheless must have been inspired to find new ways to treat disease, which was his side hustle while trying to reveal how “his” machines worked. I am sure Fred would have recognized that there is still much to be done despite all the progress made over the past several decades since these proteases were discovered. For both ClpP and proteasomes, how are proteins selected for degradation? Some clues have recently emerged: as if the PPS were not "Rube Goldberg-y” enough, Kahne *et al.* recently found a multimeric protein that specifically targets a single, essential enzyme for depupylation. This activity allows mycobacteria to fine-tune levels of the enzyme PanB in response to its product, pantothenate (100). Given that there are dozens, if not hundreds, of potential pupylated, proteasome substrates, how many other specific adaptors could possibly exist?

Neither PafA nor Dop looks anything like a ubiquitylation enzyme (65), making them potentially strong targets for drug development, given that both proteins are essential to cause lethal infections. While deletion of *dop* or *pafA* attenuates virulence in immunocompetent animals, these mutant bacilli can persist (45, 46, 63). However, it might be possible to use combinations of existing frontline antibiotics with new drugs against these PPS enzymes. Similarly, the ClpP system is essential, at least in part because of its function in ribosome rescue, but it may also regulate the functions of dozens of TA systems that likely have significant effects on *Mtb* virulence. Which TA systems, and other pathways, are affected and what is their impact on *Mtb* physiology and virulence? It is also remarkable that many microbes make inhibitors of ClpP and proteasomes, especially when the same microbes encode the proteases they inhibit (101, 102). It is likely that these molecules allow for a competitive advantage in specific environments, perhaps when a different microbial species requires its protease activity more than the inhibitor-producing strains. So much remains to be discovered, and I wish Fred were still with us to bear witness.

## Conflict of interest

The author declares no conflicts of interest with the contents of this article.
